# A Novel 3D-Printing Model Resin with Low Volumetric Shrinkage and High Accuracy

**DOI:** 10.3390/polym17050610

**Published:** 2025-02-25

**Authors:** Long Ling, Theresa Lai, Pei-Ting Chung, Sara Sabet, Victoria Tran, Raj Malyala

**Affiliations:** Glidewell Dental, Irvine, CA 92612, USA; theresalai49@gmail.com (T.L.); peggy.chung@glidewelldental.com (P.-T.C.); sara.sabet@glidewelldental.com (S.S.); victoria.tran@glidewelldental.com (V.T.); raj.malyala@glidewelldental.com (R.M.)

**Keywords:** 3D printing, additive manufacturing, model resin, shrinkage, accuracy, digital light processing (DLP)

## Abstract

This study aims to assess and compare the shrinkage, accuracy, and accuracy stability of a novel 3D-printing model resin and eight commercially available 3D-printing model resin materials. The experimental model resin was developed by our 3D-printing proprietary resin technology. Eight commercially available 3D-printing model resins were included for comparison. The AcuVol video imaging technique was used to test volumetric shrinkage. Full-arch tooth models were printed for each model resin via digital light processing (DLP) technology. The 3D average distance between the scanned model and the designed CAD digital file was applied to determine the dimensional accuracy of the 3D-printed full-arch tooth models. One-way ANOVA and Tukey’s post hoc test (*p* < 0.05) were utilized to analyze the average values of volumetric shrinkage and 3D average distance (dimensional accuracy). The experimental model resin showed significantly lower volumetric shrinkage (7.28%) and significantly higher or higher accuracy and accuracy stability (11.66–13.77 µm from the initial day to four weeks) than the other commercially available model resins (7.66–11.2%, 14.03–41.14 µm from the initial day to four weeks). A strong correlation was observed between volumetric shrinkage and dimensional accuracy (Pearson correlation coefficient R = 0.7485). For clinically successful modelling applications in restorations, orthodontics, implants, and so on, the new 3D-printing model resin is a promising option.

## 1. Introduction

The rapid development of 3D printing or additive manufacturing has brought new approaches and numerous applications in dentistry in recent years [1,2,3]. 3D printed objects have been successfully applied to modelling, implant templates, surgical guides, night guards/occlusal splints, dentures, clear aligners, and temporary restorations [4,5,6,7,8,9,10,11]. 3D printing dental model resins are the most widely used 3D printing dental materials in digital dentistry and have many applications in restoration, orthodontics, oral surgery and implants, teaching, patient consultation, and so on, for example, crown and bridge models, clear aligner models, implant models, and diagnostic models [3,12,13]. 3D-printed dental models can be constructed with more intricate geometries, more precision, and greater structural endurance than conventional dental models like plasters or stones [4,12,14]. Digital light processing (DLP), liquid crystal display (LCD), and stereolithography (SLA) are the most widely used and promising 3D-printing technologies for vat polymerization-based 3D-printed products. These technologies can achieve higher resolution and better mechanical properties for dental applications [15,16,17], as the items are printed layer by layer using photo-polymerizable resin like acrylic/methacrylic monomers. When the resins are polymerized, polymerization shrinkage is a well-known issue that can result in poor accuracy, uneven surfaces, and even warpage and curling problems [18,19,20,21]. Therefore, low-shrinkage resin material is highly desirable to obtain good accuracy in vat polymerization-based 3D-printing, as accurate and dimensionally stable dental models are the key step for the clinical success of restoration fabrication. A lot of factors affect dimensional accuracy, mainly including printing materials and their structure and composition [15,21,22,23], the printing technologies and printers used, printing parameter profile, postprocessing, and ageing [12,23,24,25,26,27,28,29,30,31,32].

Recently, we developed a new 3D-printing model resin and reported its favourable mechanical properties and degree of conversion [33]. The aim of this study is to evaluate the shrinkage, accuracy, and accuracy stability of this newly formulated 3D-printing resin and compare it to other commercially accessible 3D-printing model resin materials. The hypothesis is that the new 3D-printing model resin material has a lower shrinkage and higher accuracy and accuracy stability compared to the commercially available 3D-printing model resin materials.

## 2. Materials and Methods

### 2.1. Study Design

Nine model resins, that is, a newly developed experimental resin and eight commercially accessible model resins as comparison, were studied for volumetric shrinkage, dimensional accuracy, and accuracy stability via the DLP 3D-printing technique.

The optimal 3D-printing parameter profile for each model resin material was established by first building the depth of cure over time for each resin. The post-curing of each was followed according to the corresponding manufactures’ instructions.

The full-arch tooth model (n = 6, maximum number for Asiga 3D printer, Pro 4K UV385, Alexandria, Australia) was selected for printing for each model resin to evaluate dimensional accuracy and accuracy stability.

### 2.2. Materials

The 3D experimental model resin was prepared using our proprietary resin technology and is made up of monomers (such as ethoxylated bisphenol A dimethacrylate and urethane dimethacrylate), a photoinitiator [bis(2,4,6-trimethybenzoyl)-phenylphosphine oxide], a UV stabilizer/blocker [2-hydroxy-4-methoxybenzophenone and 2,5-bis(5-tert-butyl-2-benzoxazolyl)thiophene], an inhibitor [2,6-di-(tert-butyl)-4-methylphenol], and pigments. An overhead stirrer (IKA RW20 digital, Wilmington, NC, USA) was used to mix the resin monomers and additives for at least two hours in order to create the homogenous model resin. Eight commercially available model resin materials were selected for comparison. More information about these resin materials used in this study, obtained from the manufacturers, is shown in Table 1.

### 2.3. Volumetric Shrinkage

An AcuVol 2 instrument (Bisco, Inc., Schaumburg, IL, USA) was used to assess volumetric shrinkage (%) according to the literature [34]. Each model resin (about 15 mg) was carefully placed onto the sample holder’s base to create a bubble-free, semispherical specimen and light-cured for 20 s at a light intensity of around 1000 mW/cm^2^. Since five minutes was enough for the volumetric shrinkage to stabilize after light curing, the volumetric shrinkage of each resin material (n = 3) was measured at five minutes after light curing for twenty seconds.

### 2.4. Accuracy

The optimal 3D-printing parameter profile for each model resin material was first created based on the measurement of depth of cure (thickness over time profile) of each model resin. According to the designed digital file, the full-arch tooth models (n = 6) of each model resin were printed with an Asiga 3D printer (Pro 4K UV385) at the same layer thickness (75 µm). The 3D-printed tooth models were cleaned with isopropyl alcohol for 5 min in Form Wash (Formlabs, Somerville, MA, USA) and post-cured according to the manufacturer’s instruction. A 3Shape lab scanner (E3) (3Shape, Copenhagen, Denmark) was used to scan the 3D-printed models from above. CloudPoint Aspen software V14.2 (Aspen Tech, Burlington, VT, USA) was employed to analyze the scanned models and designed digital file. The average distance between the scanned 3D-printed model and the designed digital file was used to characterize the dimensional accuracy (trueness). The lower the average distance, the higher the dimensional accuracy. Additionally, accuracy stability was evaluated from the initial day to four weeks.

### 2.5. Statistical Analysis

Minitab 21 statistical software (Minitab, LLC State College, PA, USA) was used to conduct statistical analysis. One-way ANOVA and Tukey’s post hoc test were utilized to analyze the mean values of volumetric shrinkage and dimensional accuracy in order to assess the significance of the difference between various groups as different variables. The significance level was set at α = 0.05. Simple linear regression was used to examine Pearson’s relationship between shrinkage and dimensional accuracy for the model resin materials.

## 3. Results

**Figure 1:**
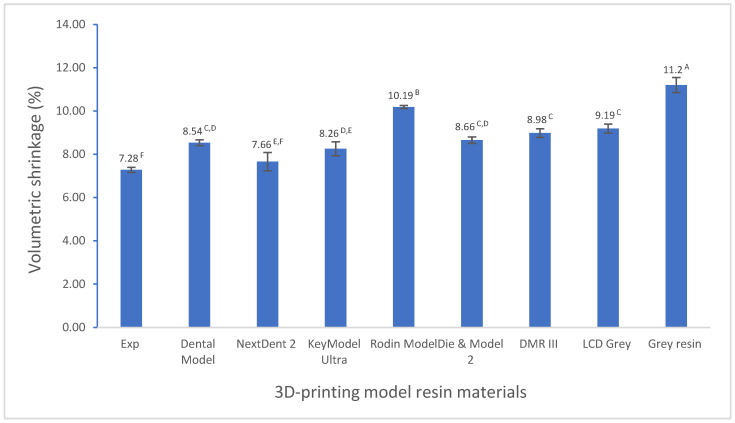
Volumetric shrinkage of 3D-printing model resin materials (values with the same superscript are not significantly different (*p* > 0.05)).

**Figure 2:**
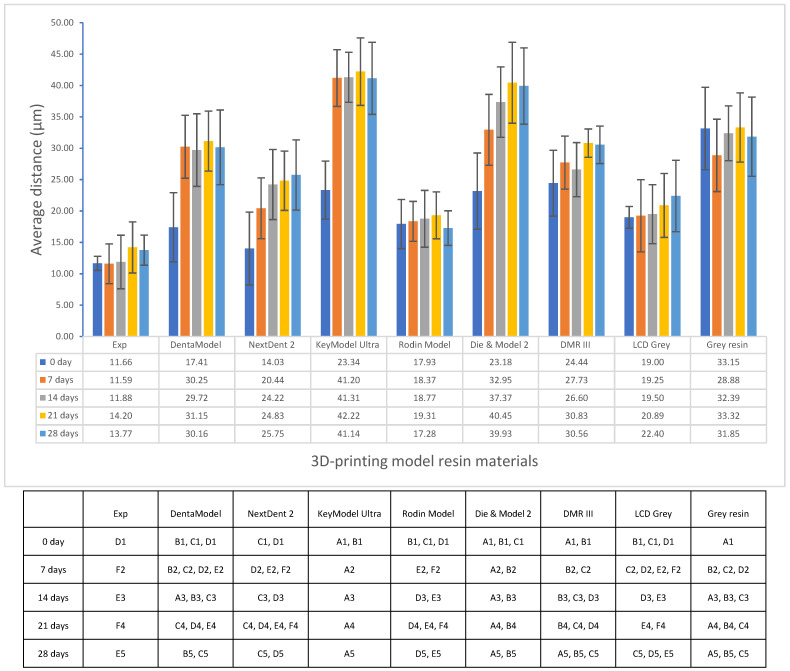
Accuracy of 3D-printing model resin materials at five measurement times (the same letters with the same number in each row of above table are statistically equivalent between the tested groups, *p* > 0.05).

**Figure 3:**
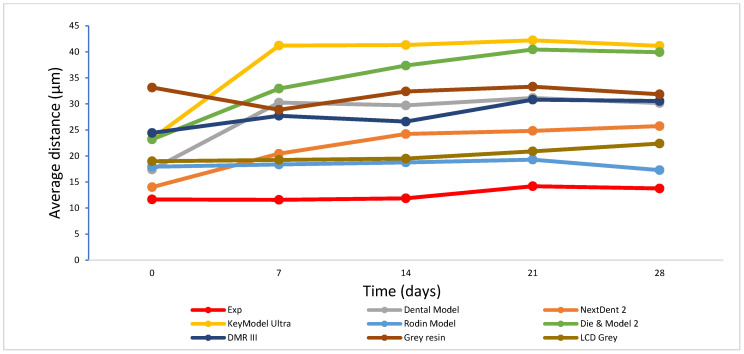
3D-printing average distances over time.

**Figure 4:**
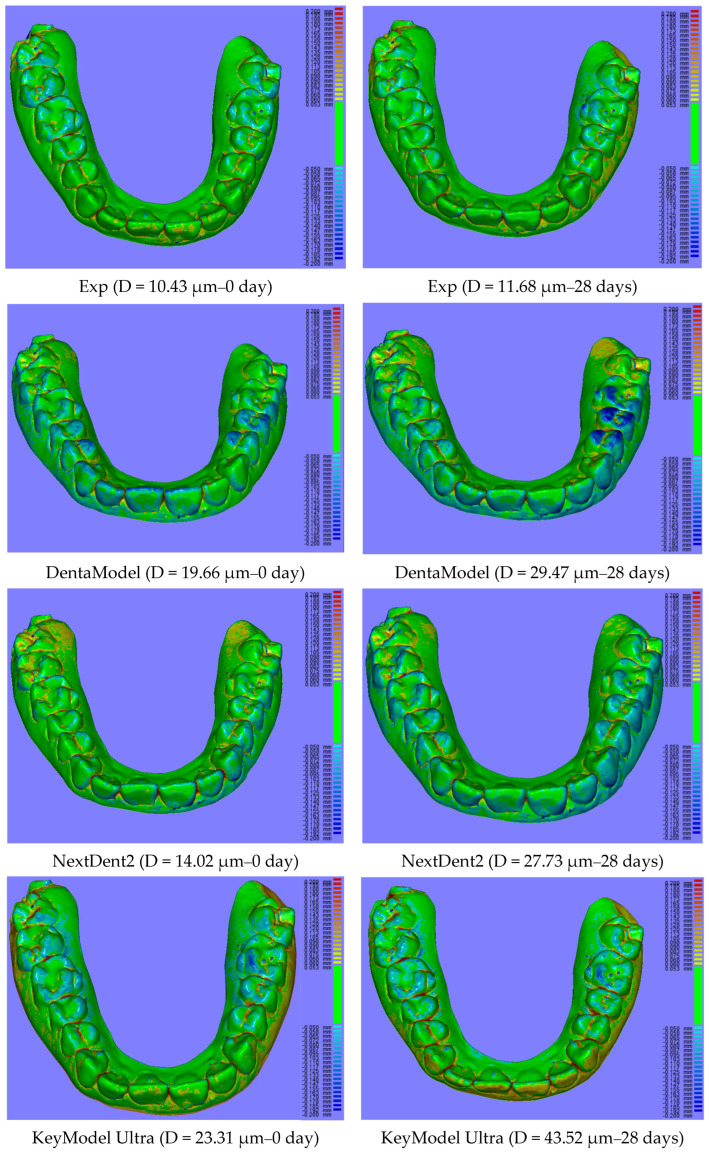

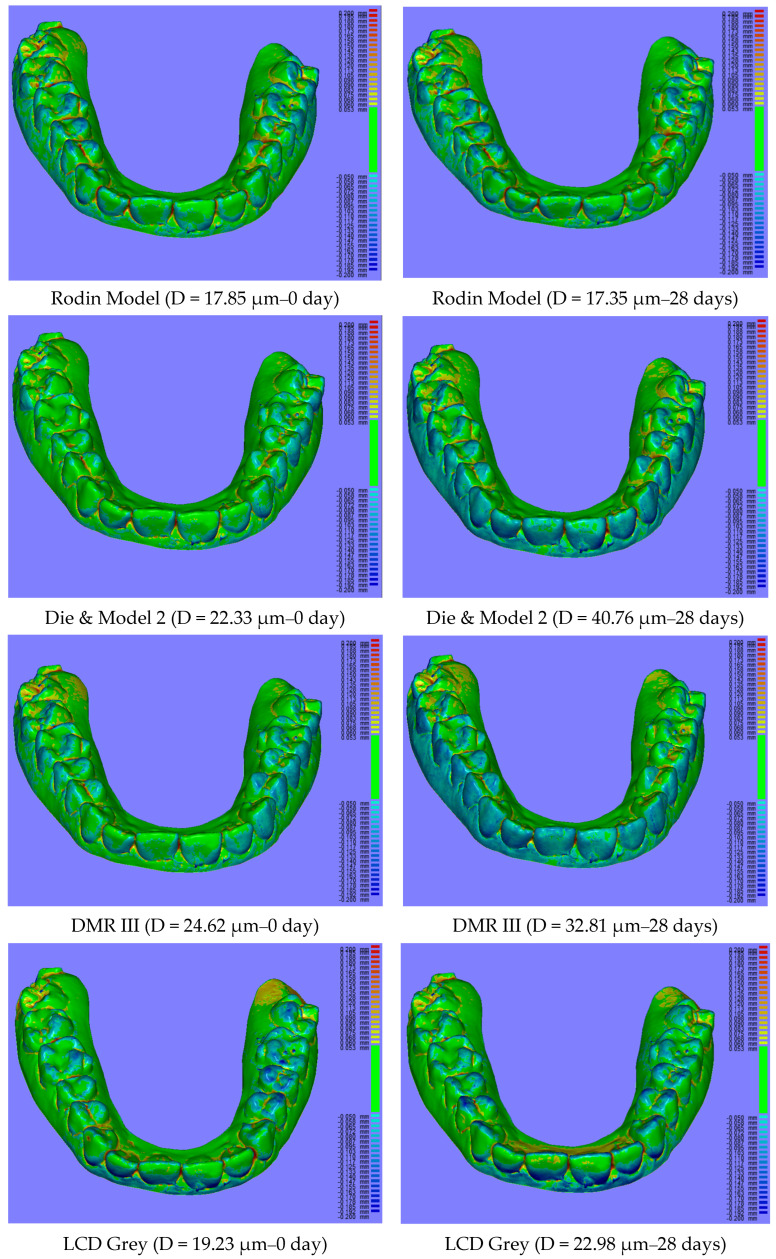

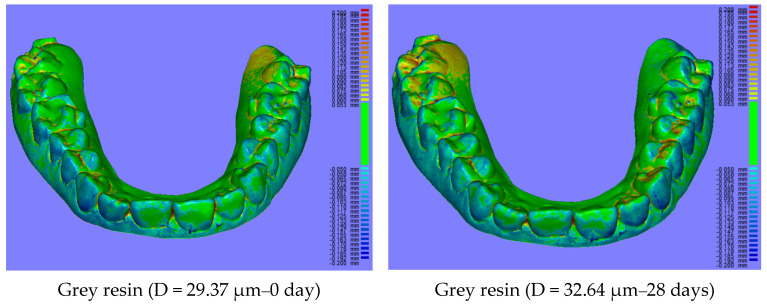
Representative images of 3D-printing average distances (D) of full-arch tooth models of resin materials at 0 day and 28 days.

**Figure 5:**
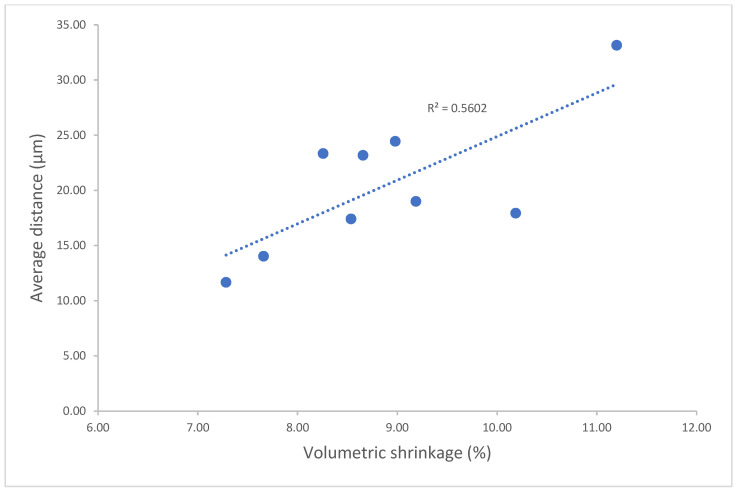
Correlation of volumetric shrinkage and 3D-printing average distances.

Significant differences in volumetric shrinkage and dimensional accuracy between the experimental model resin and the other commercial model resins were found (*p* < 0.001). The experimental model resin showed significantly lower volumetric shrinkage (7.28%) than the other model resins (7.66–11.2%) (*p* < 0.001) (Figure 1). All model resins had a lower average distance between the scanned 3D-printed full-arch model and the corresponding designed digital CAD file (<50 µm) (Figure 2, Figure 3 and Figure 4), indicating higher dimensional accuracy. However, the experimental model resin had significantly lower average distance than other model resins from the initial day to four weeks. A strong correlation with Pearson correlation coefficient R = 0.7485 (R > 0.6 or 0.7 is strong according to the Moore and Evans guidelines [35,36]) was found between volumetric shrinkage and dimensional accuracy for the initial day (Figure 5).

## 4. Discussion

Polymerization shrinkage is a well-known natural occurrence of polymerizable monomer resins during polymerization [34,37,38,39]. Because of the transformation of intermolecular forces like van der Waals into covalent bonds, shrinkage occurs when monomer molecules are transformed into polymers, resulting in a decreased free volume.

Volumetric shrinkage plays an important role in 3D printing via vat polymerization like DLP and affects dimensional accuracy and fitness for the restoration of 3D-printed objects like dental models [18,19,20,21,34]. In particular, resin materials for 3D printing are nearly pure resins devoid of fillers. In comparison to resin composites, the shrinkage may be significantly greater. This was readily apparent in the denture resin made of methyl methacrylate (MMA) monomer [40]. Therefore, for 3D-printed items, reduced shrinkage is preferred for excellent dimensional accuracy.

The AcuVol shrinkage test method is a video imaging technique that measures the volumetric shrinkage of materials before and after curing. The AcuVol software (V2) calculates and displays the relative volume change and can track shrinkage throughout the curing process in real time [41,42]. Among these evaluated model resins, the experimental resin has the lowest volumetric shrinkage value (7.28%), which is much less than that of the other resin materials (Figure 1). The difference in resin chemical structure and composition between the experimental resin and the other resin materials may explain this result (Table 1). Our proprietary resin technology, which was based on low-shrinkage monomers and also took into account the resin’s other properties such as mechanical strength and viscosity, was used to formulate the experimental 3D-printing model resin. For example, ethoxylated bisphenol A dimethacrylate with a great molecular weight and lower viscosity was employed to reduce volumetric shrinkage. In addition, triethylene glycol dimethacrylate as a diluent monomer was controlled for minimum usage, as triethylene glycol dimethacrylate will generate more shrinkage even it can increase the degree of conversion based on its chemical structure, molecular weight, and viscosity [43,44]. NextDent 2 contained methacrylic oligomers that likely have a higher molecular weight, also resulting in lower volumetric shrinkage (7.66%). DentaModel contained 7,7,9 (or 7,9,9)-trimethyl-4,13-dioxo3,14-dioxa-5,12-diazahexadecane-1,16-diyl bismethacrylate; however, it also has very-low-molecular-weight mono-methacrylate (tetrahydrofurfuryl methacrylate), which resulted in relatively higher volumetric shrinkage (8.54%) compared to the experimental resin and NextDent 2. As all manufacturers did not provide detailed information about the compositions and structures of their commercial resin materials, it is difficult to compare the differences in the resin composition and structure among these resin materials.

The quality of 3D-printed items depends heavily on dimensional accuracy, particularly when it comes to dental modelling for implants, orthodontics, and restorations. In this study, full-arch tooth models from different model resins were printed with the same printing technology and printer (DLP, Asiga 3D printer, Pro 4K UV385). As each dental model resin has a different composition and thus different reactivity to light, the printing profile of each resin will be different for obtaining better printing with good accuracy. The optimal 3D-printing parameter profile for each model resin material was first obtained based on their corresponding profiles of depth of cure over time [45], and post-curing was followed according to the manufacturer’s instructions. Therefore, the dimensional accuracy of 3D-printed full-arch tooth models mainly depends on the model resin materials themselves. Although all printed models showed lower average distances (<50 µm) against the corresponding original CAD digital file, as a dimensional accuracy of less than 100 µm is preferred [12,46], the experimental model exhibited significantly lower average distance than other resin models from the initial day until four weeks (Figure 2). This can be attributed to the difference in chemical composition and formula between the experimental model resin and the other model resin materials. As mentioned above, the experimental model resin exhibited a significantly lower volumetric shrinkage than other model resins, which made a major contribution to the lowest average distance of the experimental resin. A strong correlation between volumetric shrinkage and dimensional accuracy also supported this explanation (Figure 5). Furthermore, dimensional accuracy stability was also evaluated over time (Figure 3 and Figure 4). Some model resins like Exp. Rodin Model, DMR III, LCD Grey and Grey Resin almost showed no changes in dimensional accuracy from the initial day to four weeks, while others (DentaModel, NextDent 2, KeyModel Ultra, and Die & Model 2) indicated some increases from the initial day to one or two weeks and then became stable from one or two weeks to four weeks, probably due to the continuing polymerization shrinkage caused by the post-curing effect in the initial one or two weeks. The 3D colour-coded images enabled the visualization of the average distance between the scanned data and the CAD digital data (Figure 4) and showed the difference when the average distance was different. When the average distance was increased, some of the imaging colours changed from green to light blue or yellow and even blue or red, for example, DentaModel, NextDent 2, KeyModel Ultra, and Die & Model 2 from the initial day to four weeks. Different materials also showed some colour differences.

## 5. Conclusions

The newly formulated 3D-printing model resin displayed lower volumetric shrinkage, higher accuracy, and higher accuracy stability from the initial day to four weeks in contrast to the commercially accessible 3D-printing dental model resin materials. Therefore, the hypothesis has been proven. A strong positive correlation between volumetric shrinkage and dimensional accuracy was observed, which supported the major contribution of polymerization shrinkage to dimensional accuracy. It is expected that the new 3D-printing model resin material with high accuracy and stability has excellent clinical performance for dental modelling applications in restorative dentistry, orthodontic dentistry, implant dentistry, and so on.

## Figures and Tables

**Table 1:** The 3D-printing model resins used in this study.

Material	Manufacturer	Colour	Resin Composition
Exp. Model resin	Glidewell (Irvine, CA, USA)	Tan	Methacrylate monomers, Urethane dimethacrylate, Photoinitiator, UV stabilizer/blocker, BHT, Pigments
DentaModel	Asiga (Alexandria, Australia)	Light beige	7,7,9 (or 7,9,9)-trimethyl-4,13-dioxo3,14-dioxa-5,12-diazahexadecane-1,16-diyl bismethacrylate, Tetrahydrofurfuryl methacrylate, Diphenyl(2,4,6-trimethylbenzoyl) phosphine oxide
NextDent 2	3D System (Rock Hill, SC, USA)	Peach	Methacrylic oligomers, Phosphine oxides, Pigments
KeyModel Ultra	Keystone (Myerstown, PA, USA)	Ivory	Urethane Oligomer, Acrylate Monomers, Photo initiator, Titanium dioxide
Rodin Model	Pac-dent (Brea, CA, USA)	Pale yellow	Methacrylic Esters, Photoinitiators
Die & Model 2	SprintRay (Los Angeles, CA, USA)	Tan	Methacrylate monomers, Methacrylate oligomers, Photo-initiators, Pigments
DMR III	LuxCreo (Belmont, CA, USA)	Orange	Not available
LCD Grey	Roxel3D (Orange, CA, USA)	Grey	Methacrylate monomer(s), Photoinitiators, Pigments
Grey resin	Formlabs (Somerville, MA, USA)	Grey	Urethane dimethacrylate, Methacrylate monomer(s), Photoinitiator(s), Pigments

## Data Availability

Data are contained within this article.
